# Case of Purpura Hæmorrhagica Following "Malta" or "Mediterranean" Fever

**Published:** 1896-06-27

**Authors:** Geo. Ernest Macleod

**Affiliations:** late Assistant Physician, District Asylum, Inverness


### CASE OF PURPURA HÆMORRHAGICA FOLLOWING "MALTA" OR "MEDITERRANEAN" FEVER.

By Geo. Ernest Macleod, L.R.C.P.Edin., and L R.C S.Edin., L.F.P. and S. (Glas.), late Assistant Physician, District Asylum, Inverness.

The following account of a rapidly fatal case of Purpura Hæmorrhagica, occurring during an early period of convalescence from an acute attack of "Malta" or "Mediterranean" fever, which came under my notice a few months ago, is of considerable interest.

The patient, a naval officer, while stationed at Malta contracted a sharp attack of so-called Mediterranean fever, for which he was under treatment on the island for about seven weeks, being invalided home at an early period of convalesence by his medical attendants. When I first saw him he was in an extremely emaciated and very weak condition, having to be carried on board the steamer and being quite unable to raise himself from the horizontal position without assistance. He spoke with difficulty, and seemed to be in great mental distress, constantly brooding over his troubles and taking a very gloomy view of his condition. His face was pale and exsanguine, his tongue thickly coated and tremulous, and his bowels obstinately constipated. Radial pulse, very weak and rapid, and temperature slightly sub-normal. On his arrival on board he was immediately given a little stimulant and placed on a nutritious liquid diet of beef-tea and milk, administered alternately at short intervals, and a mixture containing iron and arsenic prescribed, the bowels being freely opened by enema. Under this treatment he improved considerably, and when seen early the next morning was found to be much brighter and more cheerful, having had a good night's rest. His pulse was now much stronger and less rapid, and his temperature normal (98·4). His face was less anæmic in appearance and his hands less tremulous, and he seemed to take much more interest in his surroundings, remarking how much better he felt, and that he thought the voyage was already doing him good. This improvement continued for two days, during which time there were no particular symptoms needing attention, the patient going on satisfactorily. On the morning of the third day, however, it was noticed for the first time that several small purpuric spots had made their appearance on his legs and forearms, and although he did not complain of feeling any worse he was decidedly weaker and less cheerful than on the previous day. Temperature 99 deg., pulse 80. Towards evening the patient had a slight attack of epistaxis, followed in about three hours' time by a much more severe one, the blood escaping freely from both nostrils in spite of the usual milder treatment, and finally necessitating plugging of the anterior nares, which succeeded in stopping it for a time. (The posterior nares were carefully examined, but no blood could be seen escaping through them.) Shortly after this a general sanguineous ooze commenced from the mucous lining of the mouth, the blood collecting round the bases of the teeth and then being swallowed and vomited up. thus simulating hæmatemesis. The patient was kept absolutely at rest in the horizontal position in a cool room, and ext. ergotæ liq. administered internally in ♏30 doses, astringent mouth washes being used frequently, which, however, seemed to have merely a temporary styptic effect.

As there was no hæmatemesis (excepting the spurious form of it above alluded to) liquid nourishment was still given by the stomach in small quantities, and iced as a precautionary measure. An interval of about two hours now elapsed, during which there was practically no bleeding at all, but after this it recommenced with redoubled severity, hæmaturia being now noticed for the first time, the blood (which was of a bright red colour) being passed towards the end of micturition, thus pointing to vesical hæmorrhage. Ergotin was now injected hypodermically, and other astringents freely administered; but in spite of all treatment the patient rapidly grew weaker and weaker, passing into an extremely anæmic state, and finally sinking from exhaustion and loss of blood.

The exceptionally malignant form of the disease, as exemplified by this case, makes it not devoid of interest, resembling as it does so closely a rapidly fatal type of hæmorrhagic purpura (*purpura fulminans*), alluded to by Roberts in the ninth edition of his "Theory and Practice of Medicine."

The pathology of the purpura which occurs during convalescence from fever is interesting. Thus Immermann (as quoted by Fagge in the third edition of his "Text-book on the Principles and Practice of Medicine,") suggests that it is possibly due to the circumstance that the recovery of the minute vessels is sometimes retarded beyond the time at which the volume of the blood is restored and the heart regains its vigour." It would be highly interesting to know in this connection whether the definite micrococcus which, according to Dr. David Bruce, is to be found in the organs in every fatal case of Malta fever, is capable of producing those changes in the blood and small vessels to which morbus maculosus is generally attributed.

